# Respiratory mucosal vaccines for emerging viruses: promise and challenges

**DOI:** 10.1128/jvi.01748-25

**Published:** 2026-07-23

**Authors:** Komal Beeton, James Brett Case

**Affiliations:** 1 Department of Virology, Immunology, and Microbiology, Boston University 1846, Boston, Massachusetts, USA; 2 National Emerging Infectious Diseases Laboratories, Boston University 1846, Boston, Massachusetts, USA; New York University Department of Microbiology, New York, New York, USA

**Keywords:** mucosal immunity, emerging viruses, mucosal vaccines, vaccines, respiratory viruses, pandemic preparedness

## Abstract

Systemically administered vaccines were instrumental in reducing severe disease, hospitalizations, and deaths during the SARS-CoV-2 pandemic. However, they often failed to consistently elicit robust immunity at mucosal sites. This minireview examines a central role for respiratory mucosal immunity in protection against emerging viral pathogens and highlights key takeaways from the SARS-CoV-2 pandemic. Evidence from SARS-CoV-2 and influenza studies demonstrates that mucosal vaccination uniquely induces secretory IgA, as well as resident memory B and T cells within the upper and lower airways, thereby promoting broader and more potent protection. Accordingly, interest in mucosal vaccination strategies has been recently renewed. However, significant knowledge gaps remain, which include determining optimal vaccination strategies (systemic prime-mucosal boost versus mucosal-only), identifying the underlying mechanisms that cause the relatively rapid decay of mucosal immunity, establishing reproducible and standardized correlates of protection, and developing safe, effective delivery platforms and adjuvants that are compatible with the respiratory environment. These challenges are particularly relevant for high-priority zoonotic threats, such as henipaviruses, hantaviruses, arenaviruses, and emerging influenza strains, for which mucosal immune responses and correlates of protection remain poorly defined. Moreover, advancing mucosal vaccine design through improved viral vectors, nanoparticle systems, and immunomodulatory adjuvants will be critical for achieving durable immunity. Ultimately, leveraging insights gained from the SARS-CoV-2 pandemic may enable breakthroughs in mucosal vaccination strategies that reduce transmission, limit viral evolution, and strengthen preparedness for future respiratory pandemics.

## INTRODUCTION

The respiratory system performs essential physiological functions necessary for survival, including gas exchange and blood oxygenation. As a result, it is a site of constant interaction with the external environment, which frequently contains potential pathogens, pollutants, or allergens. To maintain health and homeostasis, the respiratory tract comprises both physical and immunological barriers, including mucus layers, specialized epithelial cells, and tissue-resident leukocytes. The respiratory system consists of upper and lower airway compartments that contain specialized structural and immune cells responsible for maintaining tissue homeostasis while protecting against inhaled pathogens.

Given its large surface area and frequent encounters with aerosols and respiratory droplets, the respiratory tract is the primary route of entry for numerous pathogens. Prior to the emergence of SARS-CoV-2 in 2019, influenza had been the deadliest viral respiratory disease for decades (1). Nonetheless, when considered alongside diseases caused by endemic viruses such as human metapneumovirus, respiratory syncytial virus, parainfluenza viruses, or bacteria such as *Haemophilus influenzae B*, *Streptococcus pneumoniae*, and *Mycobacterium tuberculosis*, lower respiratory tract (LRT) infections are the leading cause of infectious death worldwide (2). Young children, older adults, immunocompromised individuals, and those with co-morbidities are disproportionately affected by LRT infections due to recognized immune vulnerabilities. Consequently, the effectiveness of local innate and adaptive mucosal responses plays a critical role in determining disease outcomes and in shaping immune memory.

Despite the obvious health burden imposed by respiratory viruses, research on mucosal immune responses and the development of mucosa-specific countermeasures has lagged, leaving the mechanisms required to establish, maintain, or measure effective vaccine-induced mucosal immunity largely unresolved. Potential reasons for this include challenges in tissue sampling, effective modes of delivery to mucosal sites, the requirement of high-containment facilities for many pathogens, the limited number of animal models that recapitulate human disease, and the relative ease of alternative routes (e.g., intramuscular administration). Although mucosal immune responses can be characterized in high resolution from tissues and mucosal secretions using modern techniques, this is challenging in a clinical context. To date, vaccine formulations that are compatible with mucosal administration have been restricted to live-attenuated or inactivated pathogens, which has the potential to limit their immunogenicity, durability, and/or safety (3). Addressing these challenges will be critical for the development of next-generation mucosal vaccines capable of eliciting durable and protective immunity.

Beyond endemic respiratory pathogens, sustained and increasing human-animal contact continues to elevate the likelihood of future zoonotic spillover events involving respiratory pathogens. As demonstrated by SARS-CoV-2, the capacity for emerging respiratory viruses to rapidly spread across the world is high due to global connectedness and constitutes a persistent threat to human health. The SARS-CoV-2 pandemic has provided unprecedented immunological insights into mucosal immune responses and the therapeutic potential of mucosal countermeasures. Notwithstanding, the pandemic has also shown us that respiratory mucosal immunity is highly complex and that our understanding of it is likely still in its infancy. In this minireview, we summarize some of the lessons learned over the last six years, identify key gaps in knowledge, and discuss each in the context of mucosal immunity against other emerging respiratory threats, including paramyxoviruses, bunyaviruses, and influenza viruses.

## THE RESPIRATORY TRACT AS THE FIRST LINE OF DEFENSE

**Fig 1:**
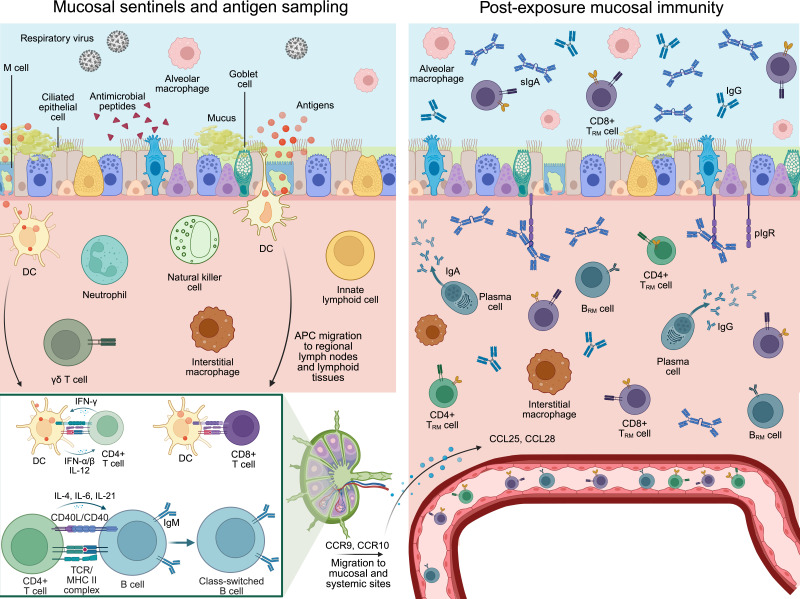
Mediators of respiratory mucosal immunity. The respiratory mucosa is lined with many highly specialized cell types that are associated with pulmonary function and protection against invading pathogens. Respiratory antigen sampling is primarily mediated by DCs and classical M cells. Following antigen uptake, DCs may migrate to regional lymph nodes or other lymphoid tissues to present antigen to B and T cells. During antigen presentation, DC-derived IFN-α/β promotes T-cell activation. Activated CD4+ T cells then facilitate activation and differentiation of B cells through CD40L-CD40 interactions and the production of IL-4, IL-5, and IL-21, while CCR9/CCR10 and CCL25/CCL28 interactions direct lymphocyte homing to mucosal tissues. IgA-secreting plasma cells and tissue-resident memory B (B_RM_) and T (T_RM_) cells are critically important for protection against subsequent exposure to respiratory pathogens.

The respiratory mucosa forms a continuous physical and immunological barrier from the nasal cavity to alveoli, protecting the host against inhaled pathogens (4, 5). Ciliated epithelial cells coordinate mucociliary clearance, while goblet cells secrete mucus that traps particles for removal (Fig. 1) (6, 7). Beyond mechanical defenses, epithelial cells produce antimicrobial peptides, mucins, surfactants, complement factors, and cytokines and act as immune sentinels that detect pathogen-associated molecular patterns through pattern-recognition receptors. In the alveoli, resident structural cells support tissue function and immune homeostasis. While these barriers are effective against pathogens, they can also limit the effectiveness of mucosal vaccines (3, 5). Following infection or vaccination, innate immune responses in epithelial and resident immune cells initiate innate antiviral responses that restrict viral replication and shape adaptive immunity. Interferons, macrophages, dendritic cells, and other immune populations contribute to pathogen control and coordinate subsequent T and B cell responses (3, 5, 8, 9).

Adaptive mucosal immunity is mediated through coordinated humoral and cellular responses. Secretory IgA (sIgA) is the dominant antibody isotype at respiratory surfaces. It contributes to protection by neutralizing pathogens, limiting epithelial attachment, and facilitating immune exclusion. However, for durable protection, cellular immunity is equally important. Following infection or mucosal vaccination, CD4+ and CD8+ tissue-resident memory T cells (T_RMs_) are strategically positioned within the respiratory mucosa and rapidly respond upon pathogen re-encounter. CD8+ T_RMs_ eliminate infected cells and suppress viral replication, whereas CD4+ T_RMs_ support local B cell responses, coordinate innate immune activation, and help maintain protective immune memory. Importantly, pulmonary T_RMs_ have been shown to protect against lethal viral challenge and can reduce infection and transmission—in some cases, even in the absence of virus-specific antibodies (5, 10–12).

The route of vaccination influences the establishment of innate and adaptive immune responses at mucosal sites (3, 5). While systemic vaccines may effectively reduce severe disease, they often do not fully prevent early replication or transmission of respiratory viruses due to limited or absent responses at mucosal sites (5, 13, 14). In contrast, emerging mucosal vaccine approaches, including nanoparticle formulations and mucosa-compatible adjuvants, aim to enhance local IgA, T_RMs_, and tissue-resident memory B cell (B_RM_) responses to potentially bridge the gap between systemic protection and sterilizing mucosal immunity.

Respiratory antigen sampling by DCs and specialized microfold (M) cells occurs in nasopharyngeal-associated lymphoid tissues (NALT), which, in humans, comprise the Waldeyer’s ring, including the tonsils (15, 16). Additionally, nasal M cells transport inhaled antigens to underlying DCs, which produce cytokines to promote T-cell activation and differentiation into Th1, Th2, and T follicular helper cells (Tfh). Activated Tfh cells subsequently promote B-cell activation and IgA class switching through cytokines such as TGF-β and IL-5 (5, 17). Following activation, for some cells, antigen uptake results in migration to regional lymph nodes, such as the cervical or mediastinal, to facilitate antigen-specific immunity (3, 5). Once activated, lymphocytes may upregulate mucosal homing receptors (e.g., CCR9 and CCR10) that direct migration to effector sites expressing CCL25 and CCL28, ultimately forming the common mucosal immune system (5).

Altogether, the respiratory tract hosts a dense and specialized cellular network composed of structural cells, resident immune populations, and dynamically recruited circulating cells that cumulatively form an integrated immune system capable of rapid detection, systemic and localized responses, and coordinated resolution following injury or infection.

## POTENTIAL LESSONS FROM THE SARS-CoV-2 PANDEMIC

The global response to the SARS-CoV-2 pandemic was unprecedented both in the scope and speed at which data were generated to characterize the virus and to develop therapeutic interventions. As the response has diminished, it is now paramount that scientists and policymakers utilize the information gained to prepare strategies against known and unknown pathogens with pandemic potential. In this section, multiple lessons from the SARS-CoV-2 pandemic are mentioned with an emphasis on their implications for future research initiatives. Notably, this list is not intended to be exhaustive, and general points are made while acknowledging that specific exceptions may exist.

### Generation of safe and effective countermeasures can be achieved rapidly

Studying viral families with pandemic potential before an outbreak occurs can provide important biological and immunological foundations that can enable a more rapid and effective response. Indeed, prior research on SARS-CoV and MERS-CoV spike proteins, coupled with emerging vaccine platforms and extensive global collaboration efforts, enabled the rapid development of safe and effective SARS-CoV-2 vaccines in less than a year. This achievement demonstrates the value of prototype pathogen research and how pandemic preparedness efforts can accelerate countermeasure development during emerging infectious disease outbreaks.

### Systemically administered vaccines do not consistently induce robust respiratory mucosal immune responses

In response to the SARS-CoV-2 pandemic, numerous vaccines were generated and tested in preclinical studies, including those that utilized novel vaccine platforms (18, 19). While systemically administered SARS-CoV-2 vaccines undoubtedly helped control the impact of the pandemic by limiting symptomatic infections, severe disease, hospitalizations, and deaths, they often failed to prevent infection or subsequent transmission (20, 21). Indeed, multiple studies have demonstrated that systemically administered vaccines elicit robust humoral and cellular responses in the blood (22, 23). However, these responses are often absent or present at very low levels in bronchoalveolar lavage (BAL) fluid obtained from humans or mice. Altogether, these findings suggest that circulating immunity may be a poor indicator for protective immunity within the respiratory tract. Similarly, in another study, a single intranasal dose of a viral-vectored SARS-CoV-2 vaccine administered to donor hamsters prevented subsequent transmission to vaccinated and unvaccinated animals, whereas transmission was unaffected by systemic mRNA-LNP vaccination (21). Collectively, these findings demonstrated that protection against infection and transmission often requires immune responses at the sites of pathogen entry and that immunity within mucosal tissues is distinct from systemic immunity. Moreover, without sufficient local mucosal immunity, initial rounds of viral replication and transmission are permitted, providing an opportunity for viral adaptation, escape from pre-existing immunity, and potential spread to more susceptible populations, which was indeed the case with the SARS-CoV-2 Omicron variant (24).

### Vaccines that reduce viral replication and transmission of respiratory viruses may improve population-level protection

In recent years, the scientific community has come under scrutiny for its response to the SARS-CoV-2 pandemic. A major criticism of SARS-CoV-2 vaccines in general is that they did not protect individuals from infection, especially following the emergence of variant strains beginning in late 2020 (24). While initially protective, vaccine efficacy against infection waned dramatically due to rapid viral evolution, and booster vaccinations failed to consistently achieve the levels of protection observed early in the pandemic. For example, a Wuhan-1/BA.5 bivalent booster reportedly had only 34% efficacy against symptomatic infection (25). Whether this reflects the timing of vaccine deployment (i.e., newly circulating variants becoming dominant before boosters can be approved and implemented widely) or the effects of prior antigen exposure on responses to variant strains remains an active area of research (26–28). Moving forward, although sterilizing immunity is an admittedly high bar, it is one that future vaccines should strive to achieve and that mucosal vaccines may be best suited. Beyond individual protection, mucosal vaccination may provide broader public health benefits by reducing transmission and contributing to herd immunity. Herd immunity occurs when a sufficient proportion of the population becomes immune, thereby indirectly protecting susceptible individuals such as those who are unvaccinated, immunocompromised, or otherwise unable to mount a protective immune response. Moreover, modest reductions in transmission can lower the effective reproductive number (R_e_) and slow outbreak growth (29). Fewer transmission events also translate into fewer opportunities for viral mutation, adaptation, and selection of immune escape variants. For rapidly evolving respiratory viruses such as influenza and coronaviruses, this public health benefit might be as important as individual-level protection. Therefore, successful mucosal vaccination strategies may not only improve protection against infection but also enhance community-level immunity, reduce overall healthcare burdens during outbreaks, and/or prevent future respiratory pandemics altogether by inhibiting replication at sites of initial entry. Accordingly, future vaccine development should prioritize strategies capable of establishing robust mucosal immunity in addition to systemic protection with the goal of improving both individual and population-level control of respiratory virus transmission.

Recent advances in respiratory vaccine development are not limited to SARS-CoV-2. Several systemically administered RSV vaccines have recently been approved, including Arexvy (GSK), Abrysvo (Pfizer), and mRESVIA (Moderna), representing an important milestone in the prevention of severe respiratory disease in older adults and other high-risk populations (30). However, these vaccines primarily aim to reduce severe disease rather than prevent infection. Nonetheless, multiple intranasal and mucosal RSV vaccine candidates are currently in development with the goal of eliciting local immune responses capable of limiting viral replication and transmission. Lessons learned from RSV vaccine development, particularly regarding vaccine durability, protection of older adults, and associated correlates of protection, may add valuable insights for future mucosal vaccine strategies against other emerging respiratory viruses.

## OUTSTANDING QUESTIONS FOR MUCOSAL VACCINES

The SARS-CoV-2 pandemic has revived interest in mucosal vaccination strategies. Currently, five mucosal SARS-CoV-2 vaccines have been approved for use in humans (Table 1), while many others are in various stages of clinical development (31). Despite this progress, several fundamental challenges continue to limit their development and widespread deployment. Although many of these concepts have been revealed through studies of SARS-CoV-2, they are broadly relevant to other respiratory pathogens. Therefore, the following unresolved questions serve as a framework for evaluating mucosal immunity and vaccine development across other respiratory virus families, which will be discussed later in this review.

**TABLE 1:** Approved mucosal SARS-CoV-2 vaccines and key clinical characteristics

Vaccine	Country	Technical platform	Approval (year)	Core clinical endpoints
Convidecia Air	China	Inhaled non-replicating Ad5-vectored vaccine administered by nebulization	2022	Serum neutralizing antibody titers, mucosal immune responses, and booster immunogenicity against circulating variants
iNCOVACC (BBV154)	India	Intranasal chimpanzee adenoviral vector (ChAd36) expressing prefusion-stabilized SARS-CoV-2 spike protein	2022	Neutralizing antibody responses, mucosal IgA induction, T-cell responses, and safety/reactogenicity
Sputnik Nasal	Russia	Intranasal heterologous adenoviral vector platform (Ad26/Ad5)	2022	Neutralizing antibody responses, mucosal immune responses, and safety
Razi Cov Pars	Iran	Intramuscular prime followed by intranasal recombinant spike protein subunit booster	2021	Prevention of symptomatic COVID-19, neutralizing antibody responses, and safety
Pneucolin	China	LAIV with deleted NS1 gene expressing the SARS-CoV-2 receptor binding domain as a secreted protein that forms dimers or trimers	2022	Protection against severe disease, prevention of symptomatic COVID-19 by variants, and safety
Convidecia Air^*a*^	Indonesia (approved as booster)	Inhaled Ad5-vectored vaccine delivered by nebulization	2022	Booster immunogenicity, neutralizing antibody responses, and safety
Convidecia Air^*a*^	Morocco (approved as booster)	Inhaled Ad5-vectored vaccine delivered by nebulization	2023	Booster immunogenicity, neutralizing antibody responses, and safety

^*a*^ The same vaccine platform (Convidecia) received regulatory authorization in multiple countries.

### What is the best vaccination approach to elicit protective mucosal immunity?

Numerous preclinical studies have assessed the effects of altering vaccine dose, timing, administration route, and/or vaccine platform to determine which strategy yields the greatest systemic protection in vaccine recipients. However, how these results translate in the context of mucosal immunity largely remains to be determined. While systemically administered vaccines are highly effective at inducing circulating antibodies and T cells that protect against severe disease, they often generate limited mucosal immunity. In contrast, mucosal vaccination induces local immune responses within the respiratory tract, including sIgA and resident memory B and T cells, thereby promoting localization and retention of protective immune responses within respiratory tissues. Accordingly, mucosal vaccination has emerged as a promising strategy for enhancing protection against respiratory pathogens. In one study, two doses of systemically administered (IM) mRNA-LNP vaccine induced circulating S-specific B and T cell immunity in the blood, but not in BAL fluid collected from the same humans or mice. However, mucosal IgA, CD8+ T cells, and lung T_RMs_ were readily detected in mucosal boosted (IN) cohorts (14, 32–34). In principle, this strategy elicits a robust systemic immune response, including the generation of antigen-specific B cells and T cells, which, upon mucosal boosting, are recruited to and retained in mucosal tissues. Moreover, intranasal vaccination against influenza or SARS-CoV-2 in preclinical models induces both local IgA and systemic IgG responses, as well as T_RMs_, providing broader protection than systemic immunization alone. Given the seemingly limited crosstalk between the two compartments, a combination of systemic and mucosal routes of vaccination may help reduce the effects of vector immunity and/or immune imprinting following the use of viral vector platforms or repeated antigen exposures, respectively, while also offering flexibility to incorporate multiple platforms.

Another challenge is immune imprinting, whereby previous exposure to related antigens recalls pre-existing immunity at the expense of generating responses to newly emerged variants. This phenomenon has been observed for both influenza viruses and SARS-CoV-2 and may contribute to reduced vaccine effectiveness against antigenically drifted strains. In some studies, mucosal boosting has been proposed as one strategy that may partially overcome this by recruiting antigen-experienced B and T cells to the mucosal tissue and simultaneously promoting local germinal center reactions and generation of *de novo* mucosal immune responses that counter the effects of immune imprinting. Moreover, mucosal vaccination also elicits sIgA and T_RMs_ that frequently recognize more conserved epitopes, therefore potentially providing better protection against antigenically drifted viruses. While additional studies are needed, these observations suggest that combined systemic prime-mucosal boost approaches may mitigate some of the effects of immune imprinting while enhancing mucosal protection.

### What are the underlying mechanisms that drive the accelerated decay of mucosal immunity?

A comprehensive understanding of the biological mechanisms that dictate this phenomenon remains a major gap in knowledge for the field of mucosal immunology. Breakthrough infections of SARS-CoV-2 are associated with reduced levels of IgA and virus-specific T cells in the URT, which, in both cases, typically wane below protective thresholds within 3–9 months post-infection (35, 36). In contrast, systemic T cells may persist for years in the blood (37, 38). A known contributor to abbreviated mucosal immunity is the half-life of IgA. While the half-life of human IgG is estimated to be approximately 3 weeks, for IgA, it is roughly only 5–6 days, which effectively decreases the protective potential of a given antibody-secreting cell by 3.5-fold to 4-fold (39). Lung B_RMs_ are induced following SARS-CoV-2 infection, and recent data suggest that peripheral lymph-node-primed B cells are recruited to the lung following an intranasal booster (33). However, questions remain regarding the best approaches to elicit these recall responses and how long mucosal memory B cells are maintained. Similarly, nasal SARS-CoV-2-specific CD8+ T cells wane faster than their systemic counterparts, decreasing to undetectable levels within 3 months following SARS-CoV-2 infection (38). T_RM_ cell pools in the lung require replenishing from circulating effector memory T cells, which eventually become depleted (40). Apart from the differences in antibody half-life and memory cell maintenance, the respiratory mucosa provides a challenging environment for long-term immune persistence. Continuous exposure to inhaled antigens drives immune responses that alter the respiratory tissues. To prevent irreversible damage, the mucosa relies on physical barriers, clearance mechanisms, and immune tolerance, which can collectively prevent prolonged maintenance of resident immune cell subsets. Therefore, strategies designed to improve durability, including periodic mucosal boosting, persistent antigen delivery systems, optimal adjuvants, and vaccine platforms that promote long-lived T_RM_ and B_RM_ populations, require further investigation. Other poorly understood confounding factors include the effects of age, genetics, and chronic respiratory diseases. Ultimately, defining the mechanistic underpinnings that dictate the decay of mucosal immunity will inform strategies to extend the protective potential of mucosal countermeasures.

### What are the correlates of protection for mucosal vaccines?

Before answers to this question can be established, it is necessary to consider the desired end goal of mucosal vaccination and the definition of “protection.” Is the objective to prevent onward transmission, severe disease, death, or to achieve sterilizing immunity? Compounding this issue is the likelihood that mucosal correlates of protection may depend on the specific pathogen and vaccine platform (or combination of platforms) used. For example, during SARS-CoV-2 infection, viral loads, pro-inflammatory cytokines, and antibody titers positively correlate with disease severity and inversely with favorable health outcomes (41). In contrast, viral loads in respiratory samples obtained during influenza infection do not consistently correlate with ongoing disease severity and are not a reliable predictor of outcome (42). In addition, the effects of pre-existing immunity, disease relevance of the animal models utilized, mucosal sampling methods, and our understanding of what constitutes protection at relevant mucosal surfaces remain incomplete for many respiratory pathogens. Nonetheless, T_RM_ cells are highly effective at restricting viral replication, and many animal studies indicate that T_RM_ levels in the lung are often a better indicator of protection than circulating memory T cells (8, 43, 44). Pulmonary T_RMs_ have been shown to protect against lethal viral challenge and can reduce infection and transmission, even in the absence of virus-specific antibodies (5, 10–12). Therefore, future efforts to establish mucosal correlates of protection should consider both humoral and cellular immune parameters. Nonetheless, potential correlates may include mucosal sIgA levels, B_RMs_, CD4+ and CD8+ T_RM_ frequencies, and functional indicators of viral control at entry sites. The relative contribution of these immune components can differ among different pathogens and vaccine platforms. Therefore, establishing standardized sampling approaches will be crucial for precise comparison of mucosal vaccine candidates and guiding the development of next-generation respiratory vaccines.

### What are the safest and most effective strategies for delivering respiratory mucosal countermeasures?

Despite the large number of pathogens that exploit mucosal barriers to infect humans, prior to SARS-CoV-2, only eight mucosal vaccines existed—of which, just one targeted a respiratory virus (3). Factors contributing to this shortage include the unique cell types and specialized architecture present at mucosal surfaces, which involve mucus layers, extracellular enzymes, and mucociliary clearance mechanisms. Thus, one of the greatest challenges for mucosal vaccine development is effective delivery. Although mRNA-LNP vaccines were instrumental in mitigating the impact of the SARS-CoV-2 pandemic, standard formulations are not compatible with the respiratory environment, resulting in mRNA degradation and poor penetration across the mucosal epithelium (5, 45). To address these hurdles, alternative nanoparticle formulations are in development, including cationic polyethyleneimine-based and inhalable, biodegradable polyamine-co-ester polyplexes (5, 46, 47). In addition, systemically administered nanoparticle formulations that traffic to specific mucosal tissues via the circulation have been generated (48, 49). Collectively, optimization of these approaches may extend the benefits of nanoparticle-encapsulated nucleic acid vaccines to mucosal sites.

Compared to existing nanoparticle vaccines, live-attenuated viral vaccines naturally penetrate the mucosal barrier. However, to ensure safety, they are often highly weakened and may still be unsuitable for very young, older, immunocompromised, or pregnant individuals (50). Therefore, the degree of attenuation must be delicately balanced to preserve immunogenicity while maintaining safety. Moreover, age-associated immunosenescence diminishes the production of sIgA, and the formation and maintenance of T_RMs_ decline, thereby weakening the first line of defense and restricting the generation of long-term local immunity. Similarly, immunocompromised individuals may be unable to safely receive live-attenuated vaccine platforms due to the risk of systemic infection and may require alternative approaches, such as formalin-inactivated or protein subunit formulations. Therefore, future mucosal vaccine development should not only prioritize efficacy but also evaluate platform-specific safety and immunogenicity across diverse populations. Even still, despite the enhanced safety profile of recombinant subunit and inactivated viral vaccines, they are often poorly immunogenic at mucosal surfaces unless administered with adjuvants. At present, the most frequently utilized include bacterial endotoxins such as heat-labile enterotoxin (LT) from *E. coli* and cholera toxin (CT) owing to their strong adjuvanting properties and capacity to induce sIgA (9). While highly immunogenic, toxicity and potential off-target effects have limited their widespread use. For instance, when administered mucosally, LT can accumulate in nearby neuronal tissues such as the olfactory bulb, infrequently resulting in Bell’s palsy in vaccinated individuals. To circumvent this issue, modified versions that retain the immunostimulatory properties while reducing toxicity have been generated and are currently undergoing safety trials (51–53).

In addition to pathogen-derived adjuvants, many PRR agonists, cytokine-based, and synthetic mucosal adjuvants are being evaluated (9). In particular, IFN-α represents a promising candidate based on its natural immunomodulatory role in the respiratory tract during viral infections. In mild-to-moderate cases of SARS-CoV-2, IFN-α is induced early upon infection and is transiently elevated in the circulation, leading to the control of viral replication and higher overall patient recovery rates (54, 55). Several clinically approved IFN-α formulations already exist with established safety and dosing profiles in humans (54, 56). In conclusion, the development of safe and effective mucosal adjuvants will facilitate the adaptation of multiple vaccine platforms for mucosal delivery. However, at present, this remains a critical bottleneck that requires further investigation.

Virus-vectored platforms are among the most promising for mucosal application. In agreement with this notion, four of the five currently licensed mucosal SARS-CoV-2 vaccines are based on viral vectors (31). This platform includes replicating and non-replicating viral vectors, although non-replicating types often offer the most favorable safety profile and broadest applicability across populations. In general, the advantages of viral vector vaccines include their natural tropism for respiratory cell types, high gene expression levels, and stimulation of innate and adaptive responses. Nonetheless, pre-existing immunity to viral vectors, such as vectors derived from human adenoviruses or immunity resulting from repeated vaccinations, has the potential to significantly hinder immunogenicity.

## MUCOSAL IMMUNITY AS THE PRIMARY DEFENSE AGAINST EMERGING RESPIRATORY VIRUSES

**Fig 2:**
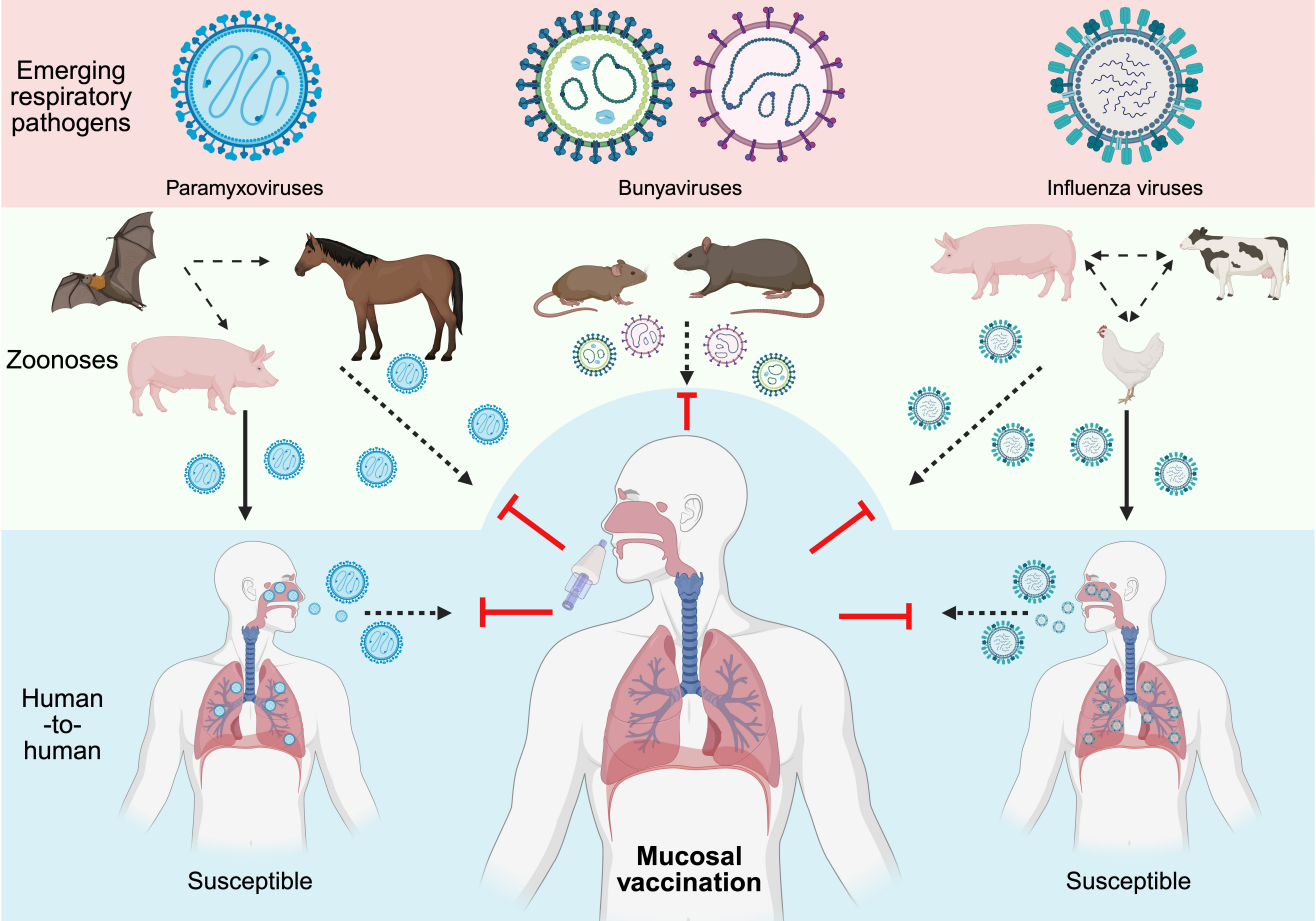
Potential protection conferred by mucosal vaccination against emerging pathogens. Respiratory viruses from multiple families circulate in animal reservoirs and pose a potential threat to human health. Upon close contact with infected animals or excreta, inhaled infectious particles can result in spillover to humans. A productive human infection may result in disease and/or spread to other individuals, potentially resulting in large outbreaks. Mucosal vaccination has the potential to restrict viral replication at initial sites of viral entry and to prevent human-to-human transmission.

Many highly pathogenic viruses are transmitted to humans via aerosols and respiratory droplets. However, only one Food and Drug Administration-approved mucosal vaccine is licensed against a respiratory virus. This is largely due to the fundamental lack of existing knowledge surrounding mucosal immune responses to natural infections or mucosally administered vaccines. In this section, we discuss the existing data, challenges, and needs for multiple viruses that pose a significant threat to human health and highlight the potential benefits of mucosal vaccination strategies (Fig. 2).

### Henipaviruses

Paramyxoviruses, a family of negative-sense, single-stranded RNA viruses, include well-established, endemic human respiratory viruses, such as measles and mumps, as well as numerous zoonotic pathogens that circulate in mammals, including Nipah (NiV) and Hendra (HeV) within the *Henipavirus* genus. Because human data remain limited, henipaviruses (HNVs) highlight many challenges in defining correlates of protection and developing safe mucosal vaccine strategies. Due to their high virulence and the scarcity of approved countermeasures, the *Paramyxoviridae* family has been prioritized by the WHO, CDC, and the Coalition of Epidemic Preparedness Innovations and was recently listed as a major concern in the NIAID Pandemic Preparedness Plan (57, 58). Since their discovery in the mid-to-late 1990s, both HeV and NiV have repeatedly spilled over into humans and/or animals, causing acute encephalitis and respiratory illness leading to death in over 50% of human infections (59). The threat of HNVs on human health is best exemplified by their (i) broad host species tropism range, spanning at least six mammalian orders; (ii) global distribution, with documented outbreaks or serological evidence of human infection in Australia, South/Southeast Asia, and Africa and animal infections on five different continents (59, 60); (iii) observed human-to-human transmission through contact with bodily fluids or respiratory droplets (61–63); and (iv) the number of newly identified HNVs, more than 17 new species described since 1994 (60, 64). One recently discovered HNV, Langya virus (LayV), was first detected and isolated from a throat swab sample collected from a febrile patient in China (65). A retrospective investigation revealed dozens of other LayV-positive patient samples. To date, no known deaths have been attributed to LayV infection. However, the true number of infections associated with this virus is largely speculative, primarily due to the recent nature of LayV recognition, overlap of symptoms with other prevalent pathogens, and the absence of virus-specific surveillance programs and diagnostics. To summarize candidly, the emergence of LayV was a near miss. Fortunately, LayV did not cause severe disease, nor is there evidence that it spread directly between humans. Like many newly discovered HNVs, the LayV fusion (F) and attachment glycoproteins (G) are highly divergent compared to their NiV and HeV counterparts, each exhibiting only ~20% sequence identity (60, 66). Although neutralizing antibodies are protective against henipaviral infections in animal models and humans (67), preliminary data indicate that the NiV and HeV countermeasures currently in various stages of clinical development do not cross-react with LayV (66). Moreover, data suggest that LayV utilizes a distinct, yet unknown, host cell receptor compared to NiV and HeV. In the setting of a true impending pandemic, absence of this knowledge would likely delay development and testing of virus-specific countermeasures significantly, especially if standard animal models were unable to support infection. Hence, how might an emphasis on mucosal immunity against emerging paramyxoviruses offer better protection going forward?

Unlike LayV, human-to-human NiV transmission via respiratory droplets has been well-documented in Bangladesh and India, especially during the Kerala outbreak of 2018 (59, 62, 63, 68–71). In principle, vaccines that elicit mucosal protection could be administered proactively to individuals at risk of contact with NiV-infected animals or individuals, or exposure to NiV-contaminated fruit (e.g., those in NiV endemic areas, agricultural workers, animal handlers, and health care workers) to decrease the likelihood of zoonotic events. However, in the event of an outbreak, like the one recently reported in Bengal (72), mucosal vaccines could be deployed more broadly to prevent further human-to-human spread.

Owing to the high virulence and mortality rates associated with NiV infection, numerous vaccine candidates have been developed, including those utilizing live-recombinant virus vectors, protein subunit, virus-like particle, and nucleic acid-based platforms (59). However, mucosal administration of HNV vaccines has been reported for only two candidates to date (73). Most notably, a virus-derived replicon particle lacking the F gene (NiVΔF VRP) was recently described (73). Following a single intranasal dose, NiVΔF VRPs elicited appreciable anti-N, anti-F, and anti-G antibody binding titers in the serum, including IgG and IgA subtypes. Interestingly, humoral responses did not exhibit neutralizing activity but rather Fc-mediated effector functions, including antibody-dependent complement deposition and antibody-dependent phagocytosis. Nevertheless, NiVΔF VRPs protected hamsters and interferon alpha/beta-receptor knockout mice from lethal NiV challenge. In contrast to NiVΔF VRP, neutralizing responses are readily detectable in serum for many of the systemically administered HNV vaccine candidates in development, but none have evaluated these responses within mucosal tissues (59). Therefore, the implementation of these countermeasures should be met with caution. As demonstrated by SARS-CoV-2, systemic responses alone can culminate in limited protection of the respiratory tract, resulting in virus replication under non-sterilizing conditions and potentially the selection of immune escape variants.

While advances in HNV research have improved our understanding of these pathogens, several critical knowledge gaps continue to limit the rational development of mucosal countermeasures. Therefore, future studies should address the following: (i) defining the correlates of protection within mucosal tissues and determining the relative contributions of humoral and cellular arms of systemic and mucosal immunity, especially that of T_RMs_, on protection; (ii) identifying the cellular receptors and host factors that dictate tissue tropism and transmission for recently described HNVs, such as LayV; and (iii) determining the durability and cross-reactivity of mucosal immune responses to infection or vaccination. Collectively addressing these gaps will improve our preparedness against emerging and pre-emergent HNVs.

### Hantaviruses

The order *Bunyavirales*, which comprises segmented, negative-sense RNA viruses, includes five virus families: *Arenaviridae*, *Hantaviridae*, *Nairoviridae*, *Peribunyaviridae*, and *Phenuiviridae*. Among these, arenaviruses and hantaviruses are transmitted to humans via aerosols, and thus, for the purposes of this review, will be the only two discussed.

Hantaviruses are globally distributed and responsible for more than 150,000 cases of disease annually (74). Hantavirus-induced illnesses range from hemorrhagic fever with renal syndrome (HFRS) in Eurasia to hantavirus cardiopulmonary syndrome (HPS/HCPS) in the Americas, with associated mortality rates between <1% and 15% and 20% and 40%, respectively (75). Hantaviruses are transmitted to humans by inhalation of contaminated aerosols derived from urine or feces of various rodent species. At present, while whole-virus inactivated vaccines against Hantaan and Seoul viruses are licensed in the Republic of Korea and China, there are no U.S. FDA-approved vaccines or therapeutic treatments for hantavirus infections (75). Instead, the response to infection consists of supportive care. In humans, it is generally accepted that HFRS and HCPS are primarily driven by vascular permeability, uncontrolled inflammatory cytokine expression, and immune-mediated pathology (76). Furthermore, while the type I interferon response is an established restriction factor for hantavirus infection and species tropism, initial suppression of the response during human infection likely contributes to uncontrolled pro-inflammatory responses, a correlate of disease severity and poor outcomes (76). This type of disease progression is reminiscent of human coronavirus infection by SARS-CoV, SARS-CoV-2, and MERS-CoV, wherein viral antagonism of the type I interferon response exacerbates disease and drives pathogenesis mediated principally by inflammatory cytokines such as TNF-α, IL-6, and IL-1 during the later stages of infection (77).

Numerous vaccine platforms and strategies have been evaluated against hantaviruses, including recombinant proteins, virus vectors, VLPs, and nucleic acid-based approaches (76). At present, data suggest that protection against lethal hantavirus infection may be achievable through multiple mechanisms. In humans, early neutralizing antibody responses are associated with favorable outcomes against HCPS-causing hantaviruses, and higher neutralizing antibody titers are observed in survivors compared to those who succumb to disease (78). In support of these observations, recombinant human mAbs administered through passive immunization are highly protective in animal models (79, 80). Moreover, in a lethal Syrian hamster model, the lone model that consistently recapitulates aspects of ANDV-induced human HCPS, depletion of CD8+ T cells results in no change in disease outcome or pathology, suggesting that these cells do not contribute to protection or disease (81). Conversely, there is evidence of protection in the absence of neutralizing antibodies, as immunization with virus-vectors or DNA-based vaccines encoding Gn, Gc, or NP confers complete protection against lethal hantavirus challenge despite low or undetectable neutralizing antibodies (76, 82, 83). Despite these efforts, the immune correlates of protection against hantaviruses are still largely unknown. Furthermore, mucosal vaccination routes and immune responses have not been evaluated in hantavirus challenge models for any platform to date (74). Individual hantavirus strains are closely associated with specific rodent reservoirs, wherein they do not cause discernible disease and often do not productively infect other related species (84). Thus, the development of animal models that recapitulate clinical diseases observed in humans or that allow the evaluation of hantavirus countermeasures has been a major hurdle in the field. Given that transmission from rodents occurs through aerosols and the fact that human-to-human transmission has been documented for ANDV (85–87), vaccines capable of eliciting sterilizing immunity at the port of viral entry, hence preventing infection altogether and/or limiting human-to-human spread, are the most desirable.

Many critical questions remain unanswered regarding protective immunity against hantaviruses. Therefore, future studies should address the following: (i) adapt existing or emerging mucosal vaccine platforms to incorporate hantavirus antigens; (ii) define the correlates of protection within the respiratory tract that are associated with protection against severe disease and reduced transmission; (iii) develop animal models that can accurately recapitulate the characteristics of HFRS observed in humans during infection with Old World hantaviruses; (iv) identify the host and viral factors that uniquely permit human-to-human ANDV transmission; (v) develop reverse genetics systems to facilitate mechanistic studies of hantavirus biology, including viral entry and replication; and (vi) define the durability of vaccine-induced mucosal immunity against homologous and heterologous hantavirus strains.

### Arenaviruses

Arenavirus countermeasures face many of the unresolved challenges discussed above, including optimal vaccination approaches, undefined correlates of protection, and unknown durations of mucosal immunity following vaccination or infection. Nine arenaviruses are known to cause human disease, the vast majority of which are distributed across Africa and South America. Lassa virus (LASV) and Junin virus (JUNV) have been designated as the Old World and New World prototypes for the family, respectively (88, 89). To date, these two viruses have received the most attention in arenavirus countermeasure development. Indeed, a live-attenuated JUNV vaccine (Candid #1), which is administered intramuscularly to at-risk individuals, has been available in Argentina since 1992. However, the widespread use of Candid #1 has been limited due to rare instances of genetic reversion and neurovirulence (88). For LASV, infection ranges from asymptomatic or mild disease to severe, life-threatening viral hemorrhagic fever in approximately 20% of cases (89). Indeed, behind Dengue virus, LASV is the most common cause of viral hemorrhagic fever, with current estimates suggesting approximately 100,000–300,000 cases annually (90). However, the true number of infections may be vastly underreported due to insufficient surveillance networks in some regions. At present, a licensed LASV vaccine remains unavailable, although numerous platforms have been evaluated, including recombinant proteins, virus replicons, VLPs, and multiple viral vectors (VSV, measles, adenovirus, and rabies vectors) (91–93). One of the most promising candidates utilizes Mopeia virus, a closely related arenavirus that does not cause disease in humans or non-human primates (NHPs). In this approach, the Mopeia virus genome was modified to include attenuating mutations within the ExoN domain of NP and to encode the LASV GP, forming a live-reassortant vaccine strain (94). Following vaccination, NHPs were protected against lethal LASV challenge, and this candidate is now in Phase Ia clinical trials. Moreover, a pentavalent form targeting five New World arenaviruses has been generated using the same platform (95). Despite this progress, reports on mucosal vaccination approaches against arenaviruses or the degree of mucosal immunity conferred by parenteral LASV vaccination are currently absent. Another major challenge for generating a safe and effective LASV vaccine is that the mechanisms of viral pathogenesis and protection are poorly characterized. Protection to LASV correlates more strongly with cellular responses than humoral, and following natural infection in humans, LASV strain-specific and cross-lineage T cells are elicited, which may be particularly important in geographical regions where multiple LASV lineages circulate (96). In contrast, humoral responses in humans and NHPs predominantly recognize non-neutralizing epitopes, likely due to the numerous N-linked glycosylation sites present within the LASV GPC protein, thereby providing a protective glycan shield against antibody recognition (97). Still, when neutralizing antibodies are generated, they require the prefusion GPC conformation and often target quaternary epitopes, which present additional hurdles for vaccine design.

To overcome these and other challenges, studies with LASV and other human arenaviruses should seek to address the following gaps in knowledge. (i) Establish the basic mechanisms of viral pathogenesis within the respiratory tract and characterize natural immune responses within these sites. Many LASV cases are asymptomatic or mild, suggesting natural immunity is sufficient to control some infections, but the immunological basis for this is unknown. (ii) Evaluate the protective efficacy of existing and emerging mucosal vaccination strategies against LASV. (iii) Determine whether neutralizing epitopes on GPC can be preferentially targeted. For example, despite extensive glycosylation, broadly neutralizing responses against the HIV-env V3-glycan epitope have been successfully elicited through rationally designed immunogens (98). (iv) Identify the epitopes recognized by broadly cross-reactive T cells. Collectively, achieving cross-reactive breadth against distinct strains and lineages within the same family should be an important consideration for future hantavirus and arenavirus vaccines, given (ii) the sporadic nature of their emergence and frequent geographic overlap and (ii) the inadequate healthcare infrastructure in many endemic regions, wherein a single vaccine conferring protection against multiple strains would be the most efficient.

### Influenza viruses

Influenza viruses, which are segmented, negative-sense RNA viruses that cause roughly 300,000–600,000 deaths annually, present numerous challenges to vaccine design and efficacy, including (i) antigenic drift, (ii) genetic reassortment, (iii) expansive animal reservoirs, (iv) numerous antigenically distinct strains, and (v) immune imprinting from previous exposures (99). However, prior to the SARS-CoV-2 pandemic, studies of responses to influenza virus infection or vaccination provided arguably the greatest insight into respiratory mucosal immunity. To this point, FluMist, an intranasal, live-attenuated influenza vaccine (LAIV; trivalent beginning in 2024-25), was the only licensed mucosal vaccine against a respiratory virus prior to those developed against SARS-CoV-2 (5). Although the global health impact, epidemiologic complexity, and biology associated with influenza virus infections are immense, this section will focus specifically on mucosal immunity in the context of emerging influenza strains.

Following influenza virus infection, B_RMs_ are induced and are capable of rapid reactivation into antibody-secreting cells upon reinfection (100). B_RMs_ contribute significantly to sIgA production, which has been demonstrated to protect against influenza infection through multiple mechanisms (101). Importantly, mucosal, but not systemic, immunization is required to elicit IgA in the bronchoalveolar space, which correlates with greater protection against secondary challenge against homologous and heterologous strains than circulating antibodies alone (14). Similarly, passive transfer of purified sIgA to naive mice confers protection against homologous and heterologous influenza strains (14, 102). In addition, T_RMs_ elicited post-vaccination or post-infection are highly cross-protective against heterosubtypic influenza strains (103–105). Like B_RMs_, T_RMs_ are observed in the airways only following mucosal immunization with LAIV and not with split-inactivated formulations (106). These data suggest that mucosal B_RM_ and T_RM_ generation is highly dependent on the site of antigen exposure and represents critical contributors to protection (5, 106).

Multiple emerging influenza subtypes have recently been recognized for their potential to cause human disease, including H5N1 avian influenza and H3N2 subclade K viruses. H5N1 avian influenza, which first emerged in 1997, is a highly pathogenic influenza A strain (107, 108). While typically a threat to livestock and poultry, severe human infections have occurred (109, 110). Recently, a new subclade, 2.3.4.4b, emerged in cattle populations throughout Europe and North America and subsequently crossed into humans (111, 112). At present, 71 cases, primarily associated with livestock caretakers, have been confirmed in the United States. Most cases have been mild, and no human-to-human transmission has been observed; however, the continued circulation of H5N1 viruses increases the potential for viral adaptation to human hosts. Although the U.S. government maintains a strategic stockpile of H5N1 vaccines developed against strains that circulated in the early 2000s, their efficacy against the currently circulating strains remains unclear. To combat this threat, an adenovirus-vectored, mucosally administered H5N1 vaccine was recently reported (113). Compared with intramuscular administration, intranasal delivery of H5 Texas HA was more immunogenic and elicited higher levels of systemic and mucosal IgA. Furthermore, serum IgG and IgA responses, but not T cell responses, correlated with protection against heterologous H5 challenge. While this approach warrants further study, for example, to determine protective efficacy against transmission and other H5 strains, it demonstrates promise against an emerging threat.

H3N2 influenza viruses are primarily associated with swine, but some strains are endemic to humans. Accordingly, the annual inactivated influenza vaccine formulation has included an H3N2 component since 1999. Despite this, a new H3N2 variant termed subclade K emerged in the spring-summer of 2025. Subclade K encodes eleven mutations in HA relative to the Northern Hemisphere 2025–2026 H3N2 vaccine strain, and data from vaccinated ferrets and humans suggest that elicited antibodies exhibit poor cross-reactivity to subclade K (114, 115). In such instances, mucosal T_RMs_, which exhibit a greater tolerance to antigenic drift, may become critically important for preventing disease and are known to be best induced through mucosal vaccination (116).

Although a LAIV is licensed in 36 countries for mucosal vaccination, its use is restricted to healthy, non-pregnant individuals between 2 and 49 years of age due to concerns of virulence. Although the LAIV has demonstrated efficacy against antigenically matched strains, particularly in children, its effectiveness is diminished by the continual antigenic drift of influenza viruses, a limitation shared with systemically administered inactivated influenza vaccines (117). A similar pattern was seen with systemically administered SARS-CoV-2 vaccines, which initially provided high levels of protection but became less effective as viral evolution gave rise to immune escape variants (24, 118). Influenza A HA undergoes antigenic drift at an estimated rate of 4.84 × 10^−3^ substitutions/site/year, enabling the virus to evade pre-existing antibody responses through mutations that alter key antigenic epitopes and receptor-binding properties, thus continually reestablishing susceptible populations (119). Why mucosal LAIV is less resilient against heterologous strains, which contrasts with many of the studies mentioned above, remains unclear, but may stem from over-attenuation and/or pre-existing immunity against the vaccine strain (120). Unsurprisingly, clear and reproducible LAIV correlates of protection continue to evade researchers. One possibility is that immunogenicity varies among individuals. In one study, 40 healthy adults were recruited, and detailed systemic and mucosal immune responses were assessed following intranasal administration of LAIV (50). Within hours of vaccination, IL-33 expression levels in some individuals inversely correlated with viral replication in the URT. At 72 h post-vaccination, lower viral replication led to intermediate IFN-γ and IL-6 protein expression levels, greater CD8+ T cell activation on d7, and enhanced mucosal IgA responses on d28. In contrast, low or absent IL-33 induction was associated with elevated viral replication, increased IFN-γ and IL-6 levels at 72 h post-vaccination, activation of circulating Tfh cells on d7, and increased peripheral blood IgG levels on d28. Although additional studies are needed, these findings suggest that differential control of viral replication, and thus antigen load, strongly influences the magnitude and localization of antibody responses, as well as the antibody isotype produced.

Despite decades of research on influenza, several important questions remained unanswered. Therefore, future studies should strive to (i) improve the immunogenicity and accessibility of the existing LAIV mucosal vaccine across all populations, especially older adults; (ii) determine whether the effects of immune imprinting associated with systemic vaccination influence responses to mucosal vaccination; and (iii) develop broadly cross-reactive mucosal vaccines that remain effective against antigenically drifted influenza viruses and protect against zoonotic transmission events, such as those occurring on cattle farms with H5N1.

## SUMMARY

Based on existing data, mucosal vaccines should be considered a core and preferred strategy for vaccination against respiratory viruses. Blocking virus entry at mucosal surfaces prevents downstream consequences, including sustained viral replication and evolution. In addition, blocking infection at mucosal sites reduces the likelihood of resistance developing to other countermeasures, such as mAbs or antivirals. Although eliciting sterilizing immunity is challenging, especially within an anatomical location that performs essential functions and has evolved to reduce prolonged inflammation, future research should prioritize the development of mucosal vaccine platforms while deepening our understanding of the immune responses elicited at mucosal surfaces following vaccination or natural infection.
